# SARS-CoV-2 Infection and Childhood Islet Autoimmunity

**DOI:** 10.1001/jamapediatrics.2024.6848

**Published:** 2025-03-03

**Authors:** Gregory J. Walker, Kylie-Ann Mallitt, Maria E. Craig, Pat Ashwood, Simon C. Barry, James D. Brown, Joanna Caguicla, Elizabeth A. Davis, Emma E. Hamilton-Williams, Leonard C. Harrison, Aveni Haynes, Tony Huynh, Guinevere Martin, Kelly J. McGorm, Grant Morahan, Zin Naing, Helena Oakey, Megan A. S. Penno, Andrea Sevendal, Richard O. Sinnott, Georgia Soldatos, Rebecca L. Thomson, Jason Tye-Din, Peter J. Vuillermin, Emily J. Ward, John M. Wentworth, Peter G. Colman, Jennifer J. Couper, Ki Wook Kim, William D. Rawlinson

**Affiliations:** 1 Virology Research Laboratory, University of New South Wales School of Biomedical Sciences and New South Wales Health Pathology, Sydney, New South Wales, Australia; 2 Sydney School of Public Health, University of Sydney, New South Wales, Australia; 3 Discipline of Paediatrics and Child Health, School of Clinical Medicine, Faculty of Medicine and Health, University of New South Wales, Sydney, New South Wales, Australia; 4 Adelaide Medical School, Faculty of Health and Medical Sciences and Robinson Research Institute, University of Adelaide, Adelaide, South Australia, Australia; 5 Telethon Kids Institute, Centre for Child Health Research, The University of Western Australia, Nedlands, Western Australia, Australia; 6 Frazer Institute, The University of Queensland, Brisbane, Queensland, Australia; 7 Walter and Eliza Hall Institute of Medical Research, Parkville, Victoria, Australia; 8 Department of Endocrinology and Diabetes, Queensland Children’s Hospital, South Brisbane, Queensland, Australia; 9 Centre for Diabetes Research, Harry Perkins Institute of Medical Research, The University of Western Australia, Perth, Western Australia, Australia; 10 Melbourne eResearch Group, School of Computing and Information Systems, University of Melbourne, Melbourne, Victoria, Australia; 11 School of Public Health and Preventive Medicine and School of Clinical Sciences, Faculty of Medicine, Nursing and Health Sciences, Monash University, Clayton, Victoria, Australia; 12 Faculty of Health, School of Medicine, Deakin University, Geelong, Victoria, Australia; 13 Department of Diabetes and Endocrinology, Royal Melbourne Hospital, Parkville, Victoria, Australia; 14 School of Biotechnology and Biomolecular Sciences, Faculty of Science, University of New South Wales, Sydney, New South Wales, Australia

## Abstract

This cohort study examines whether there is a temporal association between SARS-CoV-2 infection and the development of islet autoimmunity among Australian children with a first-degree relative with type 1 diabetes.

Increased incidence of type 1 diabetes (T1D) has been observed during the COVID-19 pandemic.^1^ In children who were genetically at risk, SARS-CoV-2 infection was associated with increased risk of islet autoimmunity (IA) in some longitudinal cohorts^2^ but not others.^3^ Additionally, COVID-19 may accelerate progression to clinical T1D in children with IA.^4^ We investigated the potential temporal association between SARS-CoV-2 infection and the development of IA in the longitudinal Australian Environmental Determinants of Islet Autoimmunity (ENDIA) study.

## Methods

ENDIA is a nationwide prospective cohort following up 1473 children with a first-degree relative with T1D from pregnancy, born between November 2012 and July 2020.^5,6^ ENDIA was approved nationally by the study’s lead Human Research Ethics Committee (HREC) at the Women’s and Children’s Health Network under the Australian National Mutual Acceptance Scheme and approved in Western Australia by the Women and Newborn Health Service and Child and Adolescent Health Service HRECs; written informed consent was given by parents or guardians of participants. Longitudinal serum specimens were collected from 1277 children (87% of cohort) from March 2013 to July 2024 and tested for islet autoantibodies (IA-2, IAA, GAD, and ZnT8) using enzyme-linked immunosorbent assay and radiobinding assay. IA was defined as the detection of 1 or more islet autoantibodies in consecutive study visits.

Substudy analysis of SARS-CoV-2 exposure included 888 children without IA, who attended at least 1 study visit from March 2020 to March 2023. Plasma were tested for SARS-CoV-2 nucleocapsid (N) and spike protein-specific antibodies by chemiluminescent microparticle immunoassay. Parent-reported SARS-CoV-2 infection histories were captured via text message surveys conducted between March 2022 and July 2023 (period of peak COVID-19 case numbers in Australia) (eMethods in Supplement 1). Infection was defined as the detection of SARS-CoV-2 N antibodies and/or parent-reported infection (positive rapid antigen and/or polymerase chain reaction test). Incidence rates for IA (prepandemic vs pandemic period, and pandemic COVID-19 positive vs pandemic COVID-19 negative) were compared, and analysis of the association between COVID-19 and IA was stratified by subgroup and age (eMethods in Supplement 1). Reporting followed the STROBE reporting guideline for cohort studies. Statistical analysis was performed using R version 4.3 (R Core Team 2024). Two-sided *P* < .05 was considered statistically significant.

## Results

Among 1277 children included (median [range] age of 2.4 [0-7] years at March 2020), the incidence rate for IA was 1.43 (95% CI, 1.04-1.93) per 100 person-years in the prepandemic period (43 cases) and 1.67 (95% CI, 1.27-2.16) per 100 person-years during the pandemic (58 cases) (incidence rate ratio [IRR], 1.17 [95% CI, 0.79-1.74]; *P* = .44) (Table). Of 888 participants assessed for SARS-CoV-2 exposure from March 2020, 404 (45.5%) had confirmed COVID-19, including 111 (12.5%) positive for SARS-CoV-2 N antibodies, 187 (21.1%) with parent-reported infection, and 106 (11.9%) positive in both datasets. COVID-19 preceded the development of IA in 6 participants and was detected concurrently with IA in 1 participant. Incidence rates were 1.43 (95% CI, 0.58-2.95) per 100 person-years among children with COVID-19 (7 cases) and 1.80 (95% CI, 1.30-2.41) per 100 person-years in those without prior COVID-19 (44 cases) (IRR, 0.80 [95% CI, 0.45-2.23]; *P* = .35) (Table). Stratified by subgroup (sex, human leukocyte antigen, vaccination) and age, SARS-CoV-2 infection did not significantly increase risk of developing IA (Figure, A and B).

**Table.** Incidence Rates for Developing IA Before and After the COVID-19 Pandemic Start, and With and Without Confirmed SARS-CoV-2 Infection

Variable	ENDIA cohort with islet autoantibody testing (n = 1277)		Substudy, SARS-CoV-2 exposure (n = 888)	
	Prepandemic (November 2012-February 2020)	Pandemic (March 2020-July 2024)	SARS-CoV-2 negative	SARS-CoV-2 positive
Children, No.	1235	1065^a^	888^b^	354^c^
IA, cases^d^	43	58	44	7
Person-years at risk of IA	3006.5	3466.8	2449.9^e^	488.7^f^
Incidence rate per 100 person-years (95% CI)^g^	1.43 (1.04-1.93)	1.67 (1.27-2.16)	1.80 (1.30-2.41)	1.43 (0.58-2.95)
Incidence rate ratio (95% CI) vs prepandemic [*P* value]	1 [Reference]	1.17 (0.79-1.74) [.44]	1.26 (0.82-1.91) [.29]	1.00 (0.45-2.23) [.99]
Incidence rate ratio (95% CI) vs SARS-CoV-2 negative [*P* value]	NA	NA	1 [Reference]	0.80 (0.36-1.77) [.35]

Abbreviations: ENDIA, Environmental Determinants of Islet Autoimmunity; IA, islet autoimmunity; NA, not applicable.

^a^ Includes 42 children born after March 1, 2020. Excludes 43 children who developed IA prior to March 1, 2020, and 169 children whose follow-up ended prior to March 1, 2020.

^b^ Children followed up during pandemic period; excludes 177 children that had no plasma sample available for SARS-CoV-2 serology (including 7 IA cases). All children (n = 888) were considered SARS-CoV-2 negative at March 1, 2020.

^c^ There were 404 children with COVID-19, confirmed by SARS-CoV-2 serology test or parent-reported SARS-CoV-2 infection. There were 50 cases excluded from incidence rate calculations, due to children having developed IA prior to COVID-19 (n = 14) or children having no further follow-up for islet autoantibodies after a positive COVID-19 result (n = 36).

^d^ IA was defined as detection of ≥1 islet autoantibodies at consecutive study visits.

^e^ Follow-up time of SARS-CoV-2 negative children from March 1, 2020.

^f^ Follow-up time from the date of confirmed SARS-CoV-2 infection.

^g^ Poisson confidence intervals.

**Figure.**
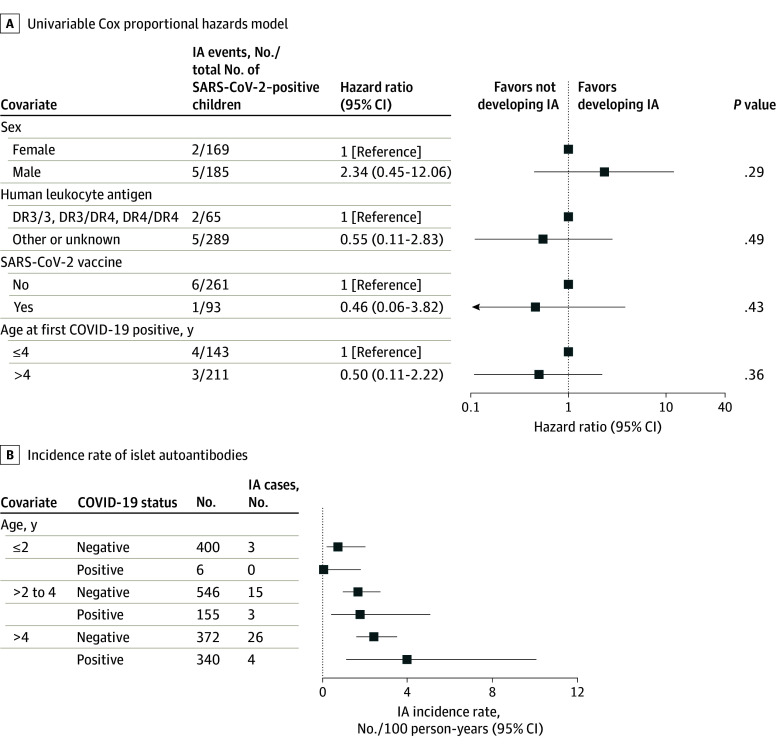
SARS-CoV-2 Infection and Development of Islet Autoimmunity (IA) in the Environmental Determinants of IA (ENDIA) Study A, Univariable Cox proportional hazards model performed with 354 children with COVID-19 who were IA negative at the time of SARS-CoV-2 detection. B, Incidence rate of islet autoantibodies from March 2020 to July 2024 in ENDIA children (n = 888), stratified by age (<2 years, 2 to <4 years, and ≥4 years) and COVID-19 status. The incidence rate at each age category was calculated for the number of new IA events observed in the interval from the preceding age category. Children have follow-up time for IA and COVID-19 occurring across multiple age categories.

## Discussion

Our findings do not support an increased risk of IA following SARS-CoV-2 infection in Australian children with a first-degree relative with T1D. This is consistent with no increased IA or T1D incidence in teens,^3^ but is in contrast to the significant temporal association observed between SARS-CoV-2 infection and IA among children 2 years of age or younger.^2^ Distinct differences between ENDIA and these Northern Hemisphere prospective studies include: (1) age of the cohorts at the time of the pandemic; (2) recruitment of ENDIA participants based on first-degree relative status, rather than high-risk T1D genotypes; and (3) the epidemiology of SARS-CoV-2 in Australia throughout the study period, which included stringent public health measures.

ENDIA is among the handful of prospective cohort studies worldwide (and the only Southern Hemisphere cohort) to longitudinally follow up children at risk for T1D for onset of IA throughout the COVID-19 pandemic. Although the small number of new IA cases occurring during the pandemic was a limitation of our study, this is characteristic of all cohorts from this period, to which our findings add valuable context to the role of SARS-CoV-2 in IA and T1D.

## Notes

Supplement 1. **eMethods.** Supplementary Materials
eReferences

Supplement 2.
Nonauthor Collaborators

Supplement 3.
Data Sharing Statement
