# Supplementary material for: SARS-CoV-2 Infection and Childhood Islet Autoimmunity
Source: JAMA Pediatr. 2025 Mar 3;179(5):568–70. doi: 10.1001/jamapediatrics.2024.6848 (PMC11877402; doi:10.1001/jamapediatrics.2024.6848)
Supplement: Supplement 1. — eMethods. Supplementary Materials eReferences [file jamapediatr-e246848-s001.pdf]

Supplemental Online Content

Walker GJ, Mallitt K, Craig ME, et al; for the ENDIA Study Group. SARS-CoV-2 infection and childhood islet autoimmunity. *JAMA Pediatr.* Published online March 3, 2025. doi:10.1001/jamapediatrics.2024.6848

eMethods. Supplementary Materials

eReferences

This supplemental material has been provided by the authors to give readers additional information about their work.

© 2025 Walker GJ et al. *JAMA Pediatrics*.

eMethods. Supplementary Materials

Environmental Determinants of Islet Autoimmunity cohort

The Environmental Determinants of Islet Autoimmunity (ENDIA) study protocol [1] and cohort profiles [2] have been published previously. Briefly, children within the ENDIA cohort (n=1473) were longitudinally followed from birth every 3 months to 2 years of age, and every 6 months thereafter until 10 years or development of T1D. Biological samples, including serum and plasma were collected longitudinally throughout follow-up. For calculation of islet autoimmunity incidence rates (pre-pandemic vs post-pandemic periods), children without islet autoantibody testing were excluded (n=196). Participants were recruited nationwide in Australia. States and jurisdictions had ranging COVID-19-related public health restrictions in place throughout 2020-2021, affecting follow-up and sample availability of some participants [3]. Thus, the sub-study of SARS-CoV-2-exposure included 888 children with available plasma for SARS-CoV-2 serology (n=177 children with no plasma were excluded), who had attended ≥1 study visits from Mar-2020 to Mar-2023 (eFigure 1A). The timing of sampling periods for SARS-CoV-2 serology and parent reported surveys are represented in eFigure 1B). The number of samples available for islet autoantibody testing and SARS-CoV-2 serology, along with median sampling intervals are presented in eTable 1.

© 2025 Walker GJ et al. *JAMA Pediatrics*.

A)

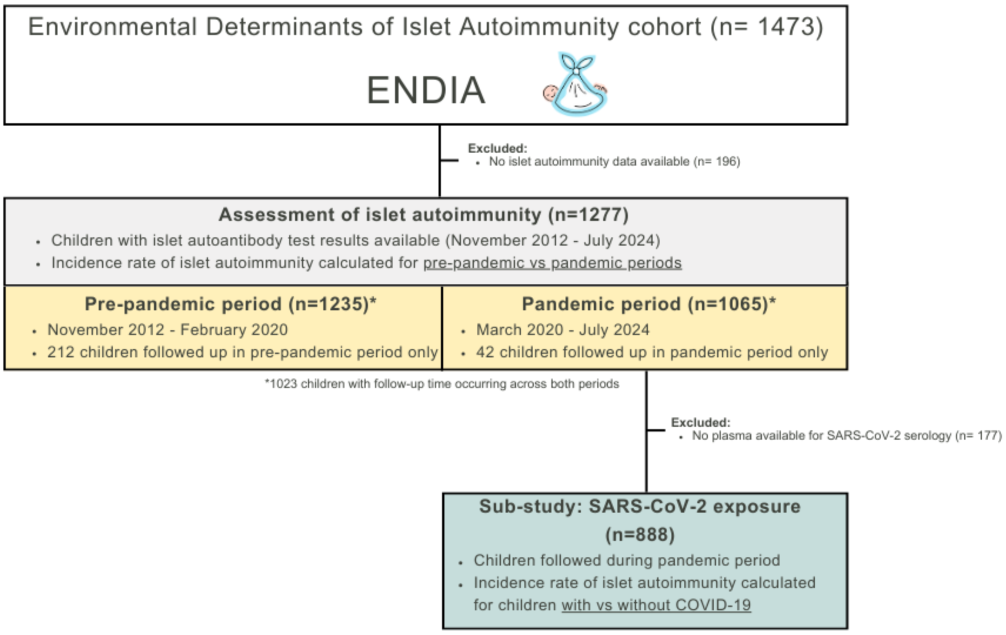

B)

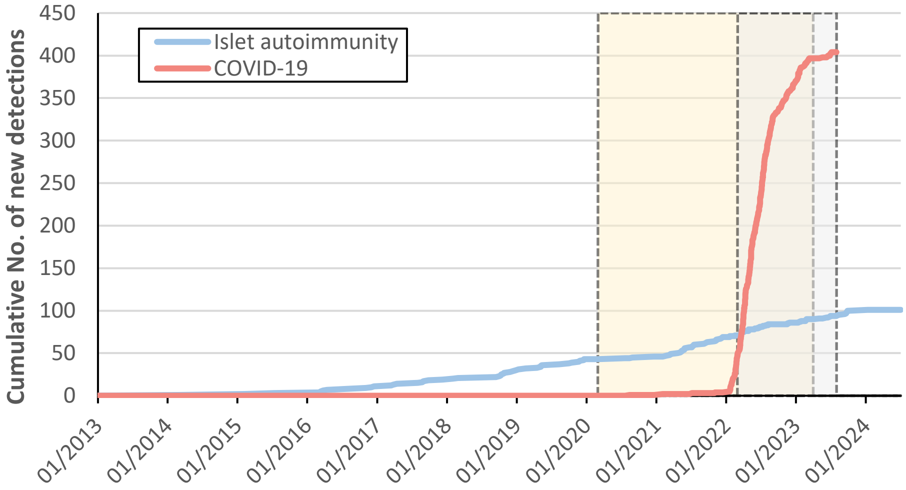

eFigure 1. The Environmental Determinants of Islet Autoimmunity (ENDIA) cohort. **A)** For children with available islet autoantibody test results, the incidence rate of islet autoimmunity (IA) was calculated for pre-pandemic and pandemic periods. Children followed during the pandemic period were assessed for SARS-CoV-2 exposure by serology and parent-reported infection histories. The incidence rate of IA was calculated for children with and without COVID-19. **B)** Sampling periods for SARS-CoV-2 serology (shaded yellow) and parent-reported infection histories (shaded grey). Lines represent the cumulative detection of islet autoimmunity (blue) and COVID-19 cases (pink) within the cohort.

© 2025 Walker GJ et al. *JAMA Pediatrics*.

eTable 1. Frequency of blood collection within ENDIA, stratified by study period and age group.

	Age group	
**Pre-pandemic summary (Nov 2012 – Feb 2020, n=1235[1])**	**≤2 years**	**>2 years**
Participants, n	1231	631
Visits, n	7421	2086
Blood collected for islet autoantibody testing, n (% of visits)	3913 (52.73 %)	1536 (73.63 %)
Median interval between collections, months (IQR)	3.35 (2.93)	6.34 (2.98)
**Pandemic summary (March 2020 – July 2024, n=1065[1])**		
Participants, n	426	1058
Visits, n	1581	6487
Blood collected for islet autoantibody testing, n (% of visits)	676 (42.76 %)	4400 (67.83 %)
Median interval between collections, months (IQR)[2]	5.75 (5.72)	6.90 (6.28)
**Sub-study: COVID-19 serology (March 2020 – July 2024, n=888[1])**		
Participants, n	362	885
Visits, n	1389	5607
Blood collected for islet autoantibody testing, n (% of visits)	638 (45.93 %)	3990 (71.16 %)
Median interval between collections, months (IQR)	3.65 (3.35)	6.54 (3.36)
Plasma available for COVID-19 serology, n (% of visits)[3,4]	561 (40.39 %)	3770 (67.24 %)
Median interval between collections, months (IQR)	3.68 (3.40)	6.64 (3.75)

[1] Unique participants in each defined period. Many children have follow-up traversing both periods and both age groups.

[2] Includes interval between pre-pandemic and pandemic collections

[3] SARS-CoV-2 serology performed to Mar-2023 only.

[4] Priority given to IA testing, where blood volume limited.

© 2025 Walker GJ et al. *JAMA Pediatrics*.

Detection of islet autoantibodies

Serum was tested for insulin (IAA), GAD, IA2 and ZnT8 autoantibodies. Autoantibodies to IAA were measured by radio-binding assay. Autoantibodies to GAD, IA2, and ZnT8 were measured by enzyme-linked immunosorbent assay with the ElisaRSR 3 Screen ICA kit (RSR Limited, UK). Assays had 100%, 98%, 100%, 94% specificity and 36%, 84%, 72%, 76% sensitivity for IAA, GAD, IA2 and ZnT8 autoantibodies respectively, in the 2020 Islet Autoantibody Standardization Program (University of Florida). Islet autoimmunity (IA) was defined as the detection of one or more islet autoantibodies, on consecutive visits at least 3 months apart. Date of IA onset was the date of first detection.

SARS-CoV-2 serology

Plasma samples were tested for SARS-CoV-2 nucleocapsid (N) and spike (S) protein antibodies by chemiluminescent microparticle immunoassay (CMIA) on the Alinity i instrument (Abbott Laboratories, USA). N-specific antibodies were detected using the *SARS-CoV-2 IgG* assay, and S-specific antibodies detected with the *AdviseDx SARS-CoV-2 IgG II* assay, performed according to the manufacturer's instructions (Abbott Laboratories, USA).

Parent-reporting of SARS-CoV-2 infection

A questionnaire was developed using Research Electronic Data Capture software (REDCap[4]) to collect additional COVID-19 data from ENDIA participants and household members. This addressed i) vaccination date/s, type, and number of doses for each child, ii) COVID-19 testing (PCR or rapid antigen test) dates and results for each member of the household (positive or negative), and iv) COVID symptoms experienced by each child. The survey was administered by a link sent via text message to the primary caregiver. Data provided by the caregiver was entered directly into the REDCap database. Eleven surveys were sent to participants one month apart from Mar-2022 until Feb-2023. Reminders were sent to non-responders weekly, with final responses received Jul-2023.

Statistical analysis

Incidence rates for islet autoimmunity were calculated as the number of events for each follow-up period, and are reported per 100 person-years with 95% Poisson Confidence Intervals. Follow-up included time until the final IA-tested sample, or the date of first islet autoantibody detection for IA-positive children. Children were considered SARS-CoV-2 negative until the date of first detection by serology or parent-reported infection. Incidence rates for IA were compared by chi-squared test. In children testing positive for COVID-19, the Cox proportional hazards model was used to analyse covariates (sex, HLA, age) associated with IA development, and vaccination status. A two-tailed p-value of 0.05 was considered significant. Data analyses were conducted using R Version 4.30.

© 2025 Walker GJ et al. *JAMA Pediatrics*.

eReferences

1. Penno MAS, Couper JJ, Craig ME, et al. Environmental determinants of islet autoimmunity (ENDIA): a pregnancy to early life cohort study in children at-risk of type 1 diabetes. *BMC Pediatr*. 2013/08/14 2013;13(1):124. doi:10.1186/1471-2431-13-124
2. Thomson RL, Oakey H, Haynes A, et al. Environmental Determinants of Islet Autoimmunity (ENDIA) longitudinal prospective pregnancy to childhood cohort study of Australian children at risk of type 1 diabetes: parental demographics and birth information. *BMJ Open Diabetes Research & Care*. 2024;12(4):e004130. doi:10.1136/bmjdrc-2024-004130
3. Penno MAS, Anderson AJ, Thomson RL, et al. Evaluation of protocol amendments to the Environmental Determinants of Islet Autoimmunity (ENDIA) study during the COVID-19 pandemic. *Diabet Med*. 2021;38(11):e14638. doi:https://doi.org/10.1111/dme.14638
4. Harris PA, Taylor R, Thielke R, Payne J, Gonzalez N, Conde JG. Research electronic data capture (REDCap)--a metadata-driven methodology and workflow process for providing translational research informatics support. *J Biomed Inform*. Apr 2009;42(2):377-81. doi:10.1016/j.jbi.2008.08.010

© 2025 Walker GJ et al. *JAMA Pediatrics*.
