# Supplementary material for: SARS-CoV-2 Infection and Childhood Islet Autoimmunity
Source: JAMA Pediatr. 2025 Mar 3;179(5):568–70. doi: 10.1001/jamapediatrics.2024.6848 (PMC11877402; doi:10.1001/jamapediatrics.2024.6848)
Supplement: Supplement 2. — Nonauthor Collaborators [file jamapediatr-e246848-s002.pdf]

Supplemental Online Content: Nonauthor Collaborators

*Indicates required information. Only first name, last name, and suffix will appear in PubMed.

*Group Name(s): ENDIA Study Group							
***First Name and Middle Initial(s)**	***Last Name**	***Suffix (eg, Jr, III)**	Academic Degrees	Institution	Location (city, state/province, country)	Role or Contribution, eg, chair, principal investigator	Group (if more than 1 Group listed in the byline) and/or Subgroup (eg, Steering Committee)
Fergus	Cameron						
Andrew	Day						
Prudence	Lopez						
Amanda J	Anderson						
William	Hu						
Dao	Huynh						
Kelly	Watson						
Sarah	Beresford						
Deborah	Bezuidenhout						
Susan	Brandrick						
Carlie	Butterworth						
Jacki	Catteau						
Helen	Griffiths						
Alison	Gwiazdzinski						
Candice	Hall						
Amanda	Hulley						
Lee	Henneken						
Renee	Kludas						
Ying	Mateevici						
Benjamin	Ramoso						
Alison	Roberts						
Alexandra	Tully						
Rosemary	Wood						
Sabrina	Binkowski						
Minh	Bui						
Abbey	Gilbert						
Dexing	Huang						
Ana	Karceva						
Brydie-Rose	Mellor						

Supplemental Online Content: Nonauthor Collaborators

*Indicates required information. Only first name, last name, and suffix will appear in PubMed.

***First Name and Middle Initial(s)**	***Last Name**	***Suffix (eg, Jr, III)**	Academic Degrees	Institution	Location (city, state/province, country)	Role or Contribution, eg, chair, principal investigator	Group (if more than 1 Group listed in the byline) and/or Subgroup (eg, Steering Committee)
Gaetano	Neselli						
Katrina	Ngui						
Trung	Nguyen						
Bina	Patel						
Vanessa	Prajitno						
Natalie	Stone						
Thao	Tran						
Sapphire	Vaega						
Yan	Xu						
Cynthia	Yau						
Rachel	Battersby						
Bek	Brittain						
Charles	Foster						
Christopher	Hope						
Preston	Leung						
Alexandra	Roth-Schulze						
Tim	Sadlon						
Bree	Tillett						
Ying Y	Wong						
Enrique	Zozaya-Valdes						
Leanne	Cavenett						
