# Supplementary material for: SARS-CoV-2 Infection and Childhood Islet Autoimmunity
Source: JAMA Pediatr. 2025 Mar 3;179(5):568–70. doi: 10.1001/jamapediatrics.2024.6848 (PMC11877402; doi:10.1001/jamapediatrics.2024.6848)
Supplement: Supplement 3. — Data Sharing Statement [file jamapediatr-e246848-s003.pdf]

Data Sharing Statement

Walker. SARS-CoV-2 Infection and Childhood Islet Autoimmunity. *JAMA Pediatr*. Published March 03, 2025. doi:10.1001/jamapediatrics.2024.6848

Data
Data available: Yes
Data types: Deidentified participant data
How to access data: Data will be made available upon request to the ENDIA Steering Committee, and approval on appropriate proposal
When available: With publication

Supporting Documents
Document types: None

Additional Information
Who can access the data: Researchers, whose proposed use of the data has been approved
Types of analyses: Validation of study analysis
Mechanisms of data availability: After approval of a proposal and with a data access agreement
